# Evaluation of the relationship between incisor torque and profile aesthetics in patients having orthodontic extractions compared to non-extractions

**DOI:** 10.1007/s00784-023-05143-7

**Published:** 2023-07-27

**Authors:** Michele Tepedino, Rosa Esposito, Maciej Iancu Potrubacz, Doniano Xhanari, Domenico Ciavarella

**Affiliations:** 1 Department of Biotechnological and Applied Clinical Sciences, University of L’Aquila, L’Aquila, Italy; 2 Department of Clinical and Experimental Medicine, University of Foggia, Foggia, Italy

**Keywords:** Aesthetic profile, Premolar extraction, Incisor torque, Borderline, Orthodontics, Biomechanics

## Abstract

**Objective:**

The aim of the present study was to evaluate the relationship between soft tissues aesthetics and incisor torque, as well as the effect of crowding, anchorage, and extraction pattern, in adult patients treated without extraction or with two or four extractions.

**Materials and methods:**

Seventy-seven subjects with permanent dentition were selected retrospectively. Among these, 24 patients were treated with four extractions, 24 with two extractions and 29 without extractions. Lateral cephalograms and photographies taken before (T0) and after (T1) treatment were retrieved. The amount of crowding and the type of anchorage were recorded, and a cephalometric analysis was performed. A one-way ANOVA was used to compare the variables within and between groups. Linear regressions were performed to evaluate the effect of different predictors on soft tissues variables at T1.

**Results:**

The statistical analysis showed no differences within and between groups for soft tissue aesthetics. A significant reduction of the angle obtained from the intersection of Frankfurt plane and mandibular plane was observed in the four-extractions group, and a significant proclination of the lower incisors was observed in the two-extraction group. Linear regressions revealed that the change in soft tissue profile aesthetics was affected by the type of anchorage and the two-extractions pattern.

**Conclusions:**

Similar soft tissue aesthetics were observed after treatment in the three groups, despite the presence of some skeletal and dental differences.

**Clinical relevance:**

A well-controlled incisor torque helps to preserve soft tissues aesthetics. The type of anchorage could influence soft tissues.

**Supplementary Information:**

The online version contains supplementary material available at 10.1007/s00784-023-05143-7.

## Introduction

The extraction of premolars for orthodontic treatment and their effects on soft tissue are highly debated topics in orthodontics. Extractions are used to solve moderate-to-severe crowding, and to alleviate dental or dentoalveolar protrusion [1, 2], and they are recommended in patients with dental and skeletal sagittal problems, open-bite, and increasing overjet [3].

It has been shown that the two most important parameters influencing the extraction or non-extraction decision are the soft tissue profile and the amount of crowding [4]. In fact, especially in borderline patients, where there are no evident indications of whether extraction is necessary, an appropriate study of patients’ frontal and profile appearance is recommended.

A recent study by Jackson et al. observed a mild decreasing trend of extraction cases, settling near 25% of total treated cases at the university clinic setting from 2000 to 2011 [3]. This change may be due to the evolution of modern orthodontic techniques [4] but also to the patients’ reluctance to have healthy teeth extracted. Another reason may be the greater importance given to facial profile in recent years. In fact, some authors concluded that extractions may straighten the patient’s profile, thus worsening its aesthetics [5, 6], although the literature also offers studies that contradict the association with negative changes of soft tissues appearance [7]. It has been shown that lips deserve an accurate pre- and post-treatment evaluation because after orthodontic extraction, the lip profile became more concave than in non-extraction patients; however, this difference was small and clinically irrelevant in most of the cases [8], and highly dependent on lip thickness [9].

Moreover, in the literature, there are many studies that analyse the effects produced by orthodontic extractions on profile aesthetics, but, to the knowledge of the authors, none of these has ever verified the correlation between the torque of incisors and the aesthetics of the profile. Indeed, previous studies were mainly focused on extractions per se, while the mechanics used and their effect on facial profile were often neglected. This last consideration should not be overlooked because it is common knowledge that the retraction of the anterior teeth is followed by a loss of torque, which leads to a more upright position of the incisors [10]. Therefore, it can be postulated that it is not the extraction treatment itself that causes profile straightening, but rather the use of incorrect space closure mechanics, which results in insufficient torque of the incisors and could consequently be the cause of profile worsening. A closer look at these aspects could provide new and clinically meaningful data in the extraction/non extraction debate.

Therefore, the aim of the present study was to evaluate the relationship between soft tissue profile aesthetics and incisor torque, as well as the effect of crowding, anchorage, and extraction pattern, in adult patients treated without extraction or with two or four extractions. The null hypothesis was that no interaction exists between the predictors and the response variables.

## Materials and methods

This retrospective study and all the procedures that followed were approved by the Ethical Committee of the University of L’Aquila (protocol no 28954, ID 04/2021) and, were in accordance with the Declaration of Helsinki from 1975 and subsequent revisions. Written informed consent was obtained from the subjects or their legal tutors.

### Patients

Sample size calculation revealed that to be able to reject the null hypothesis for a linear multiple regression, all coefficients in the model had to be equal to zero. Considering a medium effect size (f^2^) of 0.15 [11], with a power of 80% and a type I error of 5%, 77 subjects were needed for the whole sample (G*Power version 3.1.9.2, Franz Faul, Universität Kiel, Germany). Seventy-seven patients were thus selected after screening the records of patients treated at the Orthodontic Clinic, Department of Biotechnological and Applied Clinical Sciences, University of L’Aquila, in chronological order from January 2011 to December 2019, using the following inclusion criteria:

- Patients with permanent dentition;
- Class I or Class II molar relationship;
- Orthodontic treatment with extraction of two or four premolars, or non-extraction orthodontic treatment with fixed appliances;
- Lateral cephalograms taken before and after treatment;
- Complete clinical documentation concerning the photographic documentation, the study casts and the type of orthodontic treatment received, indicating the type of mechanics used to close spaces and the type of anchorage used.

The exclusion criteria were as follows:

- Patients in mixed dentition;
- Extraction of teeth other than premolars;
- Patients who have undergone orthognathic surgery;
- Presence of congenital abnormalities regarding dentition or craniofacial growth;
- Patients with cleft lip.

The study sample was divided into three groups: the first study group was composed of 24 patients treated with four extractions; the second study group was composed of 24 patients treated with two extractions in the upper arch; finally, a control group was composed of 29 subjects treated without extractions. For each patient, the pre-treatment clinical condition was evaluated, defining the Angle’s molar class and the amount of crowding. The amount of crowding in mm was calculated for each arch from the dental casts as the total tooth-size arch-length discrepancy with the method described by Nance. Then, the upper and lower arch of each patient was classified into three categories of severity based on the amount of crowding, according to the criteria proposed by Proffit [10]: mild crowding between 0 and 4 mm; moderate crowding between 5 and 9 mm; severe crowding when greater than 9 mm. In addition, the type of programmed anchorage (maximum posterior anchorage, minimum posterior anchorage, and medium anchorage) for both the upper and lower arches was extracted from the clinical history and recorded.

### Cephalometric analysis

Cephalometric analysis was carried out on digital lateral cephalograms, taken in natural head position, at two different timepoints, before treatment (T0) and post-treatment (T1).

Measurements were performed by one operator using a cephalometric software (Orisceph CE, Elite Computer Italia srl, Vimodrone, Italy). Images were appropriately calibrated using reference points located at a known distance. To perform the cephalometric analysis, a personalised cephalometric method was previously created and inserted into the software’s database.

**Fig. 1:**
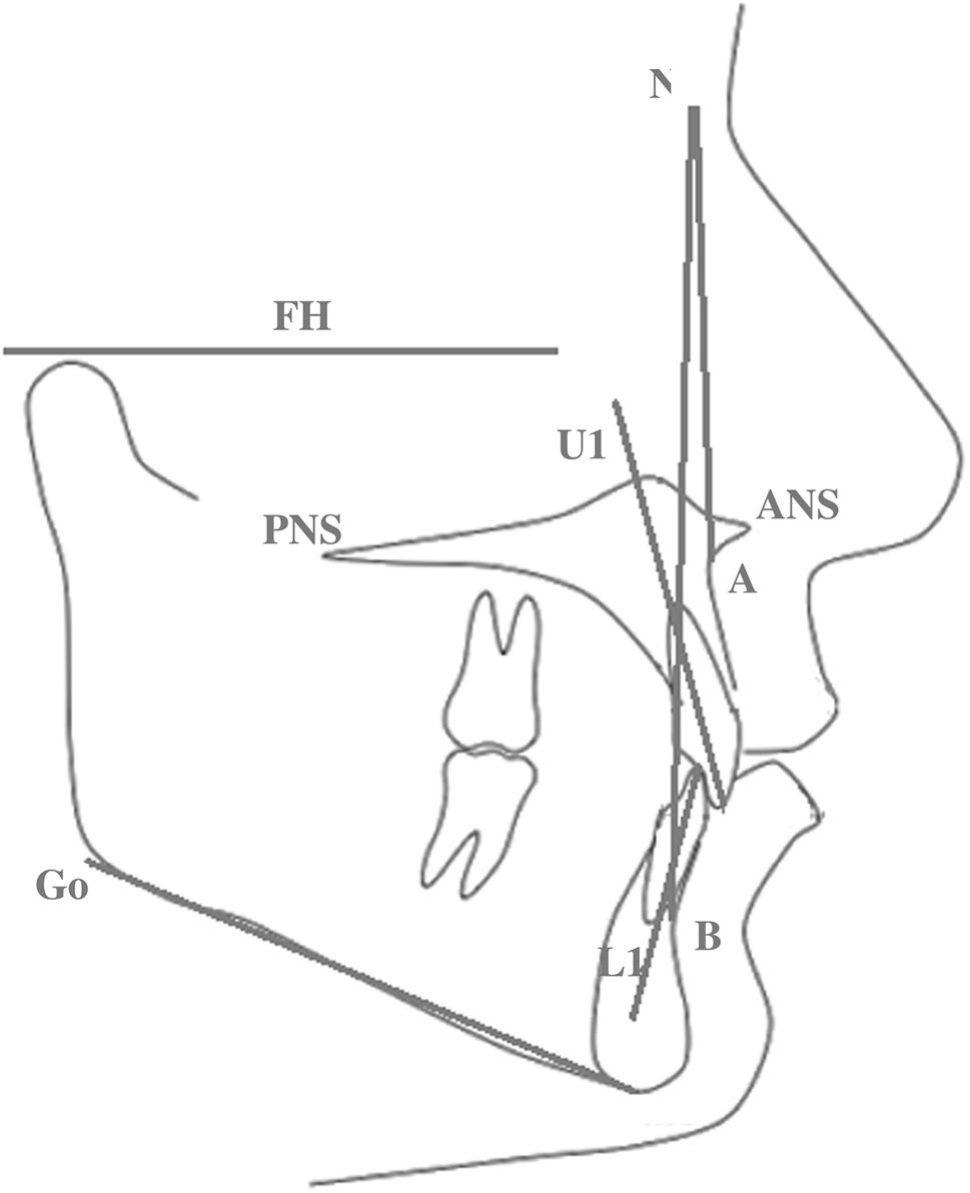
Cephalometric template used in the study for the evaluation of skeletal and dental changes. The nasion point N: the most anterior point of the front-nasal suture, FH: Frankfurt Horizontal plane, A point: the deepest point on the curvature of the maxillary alveolar process (or sub-spinal point of the maxilla), B point: the deepest point on the curvature of the mandibular alveolar process (or supra-mental point of the mandible), ANS: the anterior nasal spine, PNS: the posterior nasal spine, U1: long axis of the upper incisor, L1: long axis of the lower incisor, Me: menton point, Go: gonion point

Skeletal changes were evaluated by assessing the angle formed by the intersection of the Frankfurt plane with the mandibular plane (FMA) and the ANB angle between the A point (the deepest point on the curvature of the maxillary alveolar process, or sub-spinal point of the maxilla), the Nasion point, (N, the most anterior point of the front-nasal suture) and the B point (the deepest point on the curvature of the mandibular alveolar process, or supra-mental point of the mandible) (Fig. 1).

Dental changes were assessed by measuring the interincisal angle (the angle resulting from the intersection of two planes: one passing through the long axis of the upper central incisor and one passing through the long axis of the lower central incisor), the torque of the upper incisor (the angle between the long axis of the upper incisor and the palatal plane passing through the anterior nasal spine and the posterior nasal spine), and the torque of the lower incisor (the angle between the long axis of the lower incisor and the mandibular plane passing through the menton and gonion points) (Fig. 1).

**Fig. 2:**
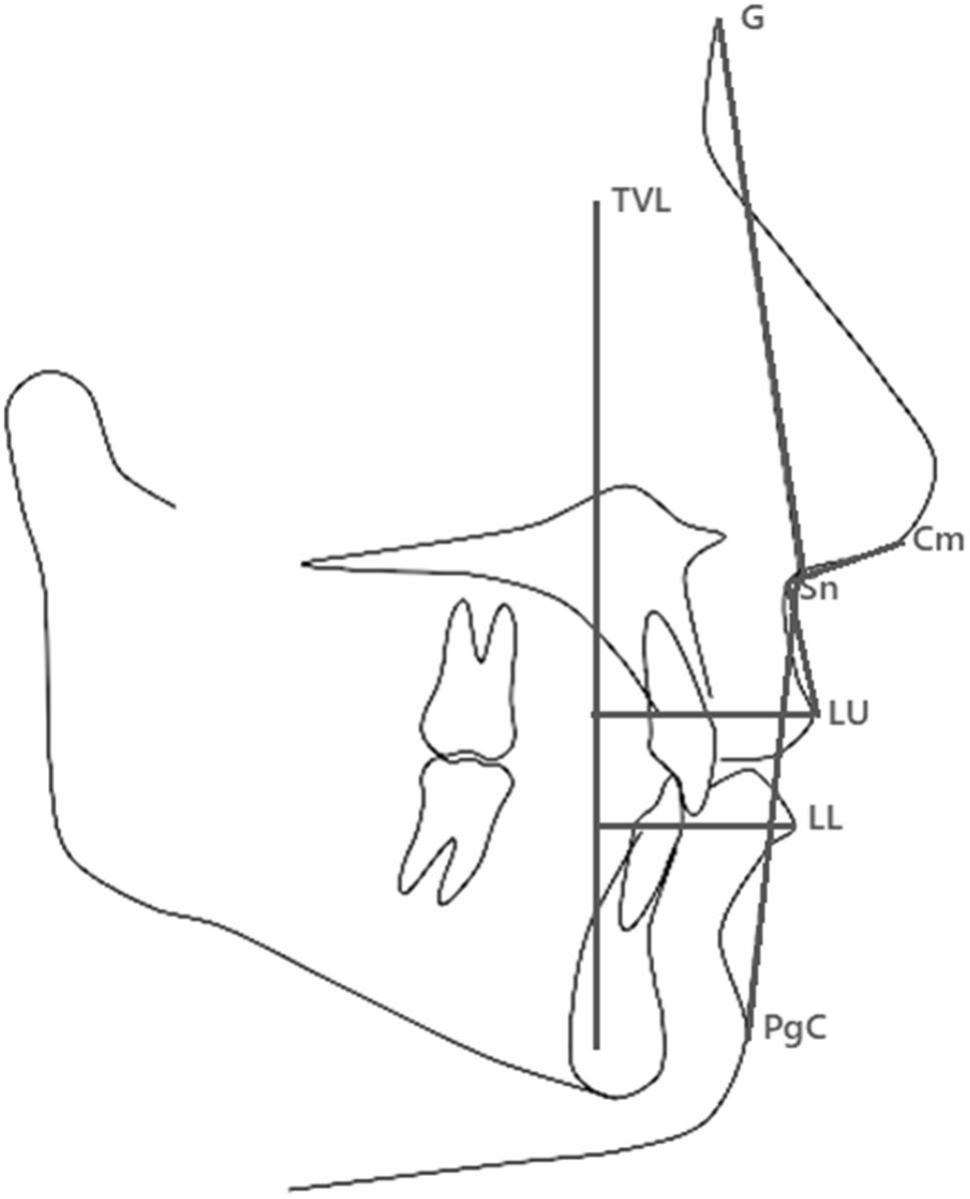
Cephalometric template used in the study for the evaluation of soft tissues changes. TVL: perpendicular line to the Frankfurt plane, LU: the most protruding point of the upper lip, LL: the most protruding point of the lower lip, Sn: base of the nose, G: glabella point, located on the foremost point of the forehead, PgC: pogonion cutaneous point

To evaluate labial changes a true vertical line (TVL) was first drawn, tracing a line perpendicular to the Frankfurt plane passing through N. Then, the orthogonal distance between the most protruding point of the upper lip (LU) and the TVL and the orthogonal distance between the most protruding point of the lower lip (LL) and the TVL were measured (Fig. 2).

Profile changes were evaluated assessing the angle of profile convexity (Facial convexity angle), which was obtained by connecting the glabella point (G), located on the foremost point of the forehead, base of the nose (Sn) and pogonion cutaneous point (PgC) (Fig. 2).

### Error of the method

To evaluate the error of the method, the radiographs of 25 subjects, selected randomly using an online tool (www.randomizer.org), were re-traced by the same operator after 2 weeks. The random error was calculated using Dahlberg’s formula [12]. The intra-rater agreement was assessed using an intraclass correlation coefficient.

### Statistical analysis

Descriptive statistics for all variables were calculated. A Shapiro − Wilk normality test was computed for all the variables to evaluate the type of data distribution. To verify that before treatment (T0), the three groups showed comparable characteristics described by the measured variables, a one-way ANOVA was performed, testing data homoscedasticity with a Levene test and using Tukey’s or a Games − Howell post hoc test to perform pairwise comparisons. Similarly, to verify that the distribution of age, gender, the type of crowding (mild, moderate, and severe) and anchorage used (minimum, moderate and maximum) were comparable between subjects belonging to the three groups, a Fisher’s exact test was performed.

To assess the presence of a statistically significant change between cephalometric variables before and after treatment, a paired *T*-test or a Wilcoxon signed rank test – − depending on the type of data distribution – − was performed for each pair of variables at T1 and T0. To evaluate the differences in the T1 − T0 changes among the three groups, a one-way ANOVA was computed.

Finally, to evaluate the effect of the extraction pattern, the amount of initial crowding, the final position of the upper and lower incisors, and the type of anchorage used on the T1 values of the variables related to the soft tissues (LU-TVL, LL-TVL, facial convexity angle), linear regressions were performed, in addition to Durbin − Watson statistics and residual analysis through q-q plots.

For all statistical tests, a type I error of 0.05 was set.

## Results

Descriptive statistics are reported in Table 1.

**Table 1:** Descriptive statistics

*Variables*		*Number of extractions* = *0*			*Number of extractions* = *2*			*Number of extractions* = *4*		
		*T0*	*T1*	*Δ (T1–T0)*	*T0*	*T1*	*Δ (T1–T0)*	*T0*	*T1*	*Δ (T1–T0)*
LU-TVL (mm)	Mean	22.02	21.83	− 0.19	15.02	14.49	− 0.53	15.49	15.82	0.33
	SD	6.36	5.76	5.35	4.08	3.86	3.86	4.64	4.13	3.95
	Normality test	0.379	0.574	0.001	0.973	0.166	0.026	0.334	0.075	0.488
LL-TVL (mm)	Mean	19.89	19.93	0.03	11.03	11.24	0.20	12.71	13.22	0.51
	SD	5.91	6.38	5.36	5.14	4.09	4.69	4.59	4.76	4.40
	Normality test	0.936	0.291	0.003	0.913	0.983	0.033	0.625	0.396	0.962
ANB (°)	Mean	2.80	2.48	− 0.32	5.45	5.05	0.40	4.40	3.73	− 0.67
	SD	2.62	2.06	1.65	2.69	2.10	1.72	2.03	1.99	1.76
	Normality test	0.029	0.010	0.013	0.735	0.097	0.609	0.001	0.020	0.216
FMA (°)	Mean	18.74	19.54	0.80	24.09	24.11	0.020	24.90	20.09	− 2.01
	SD	5.59	6.70	3.90	5.57	5.61	2.24	6.06	6.25	3.35
	Normality test	0.115	0.070	0.803	0.686	0.558	0.563	0.427	0.334	0.085
Interincisive (°)	Mean	127.1	123.89	− 3.21	134.20	128.92	− 5.28	132.15	137.10	4.95
	SD	8.75	8.27	8.98	8.21	6.94	8.90	10.02	7.05	10.66
	Normality test	0.151	0.790	0.169	0.647	0.871	0.871	0.630	0.510	0.515
L1-GoMe (°)	Mean	82.86	81.12	− 1.74	92.74	97.52	4.78	91.84	91.97	0.13
	SD	5.65	6.60	7.08	6.60	5.77	5.76	7.40	8.00	6.02
	Normality test	0.618	0.114	0.188	0.674	0.465	0.770	0.163	0.146	0.630
U1-ANSPNS (°)	Mean	110.50	112.78	− 0.18	106.54	107.25	0.65	110.16	107.47	− 2.60
	SD	6.93	6.76	6.44	6.17	5.12	5.31	6.90	5.28	6.99
	Normality test	0.485	0.464	0.107	0.978	0.501	0.570	0.315	0.453	0.553
Facial convexity (°)	Mean	151.33	151.09	− 0.24	157.90	157.70	− 2.65	161.12	162.45	1.33
	SD	4.63	5.01	3.98	8.51	7.83	5.78	6.32	4.03	3.98
	Normality test	0.593	0.491	0.834	0.415	0.233	0.02	0.816	0.621	0.263

LS-TVL: the orthogonal distance between the most protruding point of the upper lip (LU) and the true vertical line (TVL); LL-TVL: the orthogonal distance between the most protruding point of the lower lip (LL) and the TVL. The TVL was drawn tracing a perpendicular line to the Frankfurt plane passing through Na. FMA: the angle formed by the intersection of the Frankfurt plane with the mandibular plane passing through gonion (Go) and menton (Me); ANB: the angle between the A point (supra-spinal point of the maxilla), the nasion point (Na), and the B point (submental point of the jaw). Interincisal angle; the angle resulting by the intersection of the plane passing through the long axis of the upper central incisor and one passing through the long axis of the lower central incisor; U1-ANSPNS: the inclination of the upper incisor the angle between the long axis of the upper incisor and the palatal passing through ANS and PNS; L1-GoMe: the inclination of the lower incisor results of the angle between the long axis of the lower incisor and the mandibular; facial convexity: the angle obtained by connecting the glabella point (G), located on the foremost point of the forehead,Sn and pogonion cutaneous point (PgC)

The random error measured with the Dahlberg formula for linear measurement ranged between 0.23 and 0.48 mm, while the random error for angular measurement ranged between 0.32 and 0.39°. The intra-rater agreement, assessed through an intraclass correlation coefficient, was above 0.9 for all variables.

**Fig. 3:**
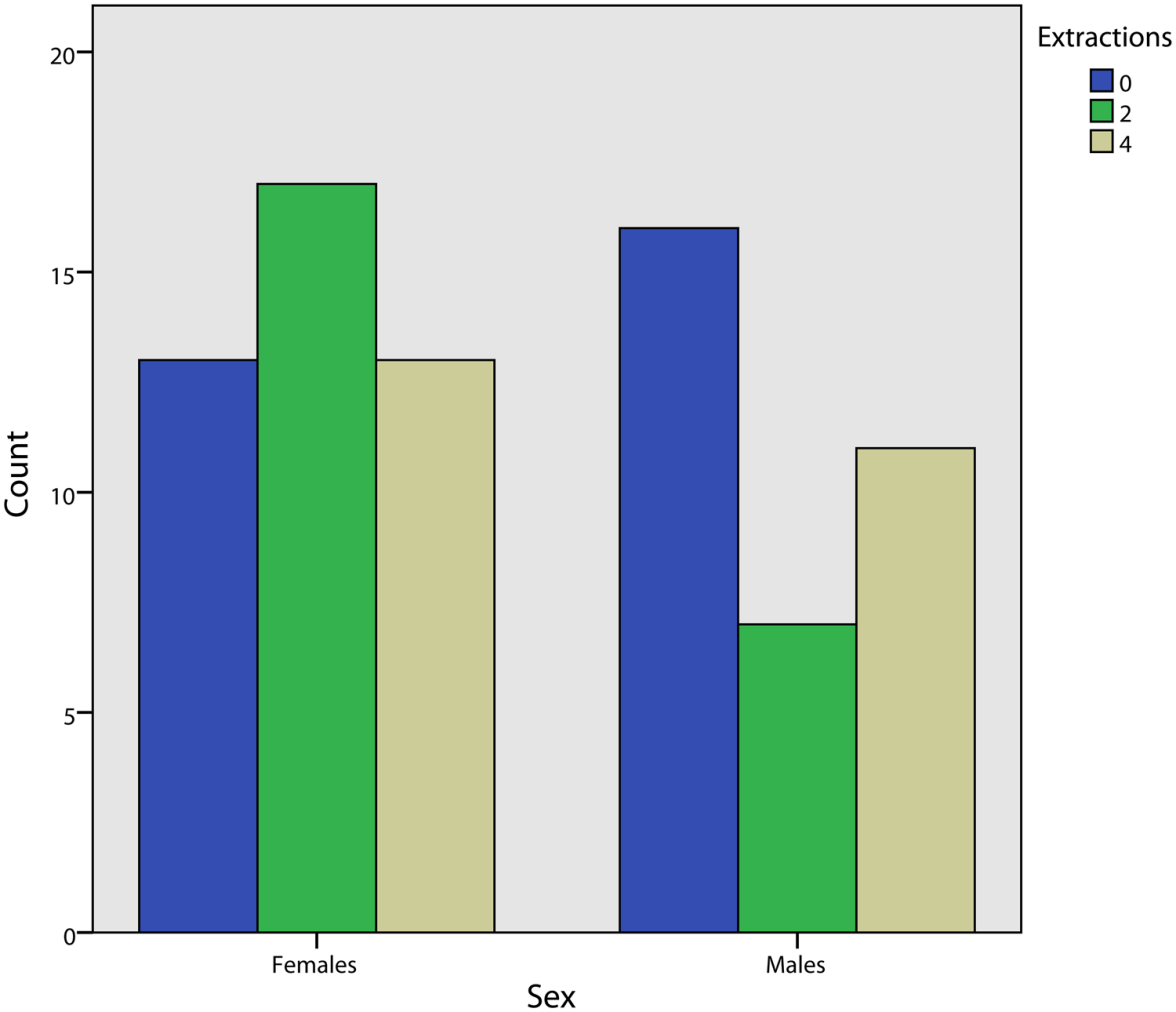
Distribution of sex in each group

**Fig. 4:**
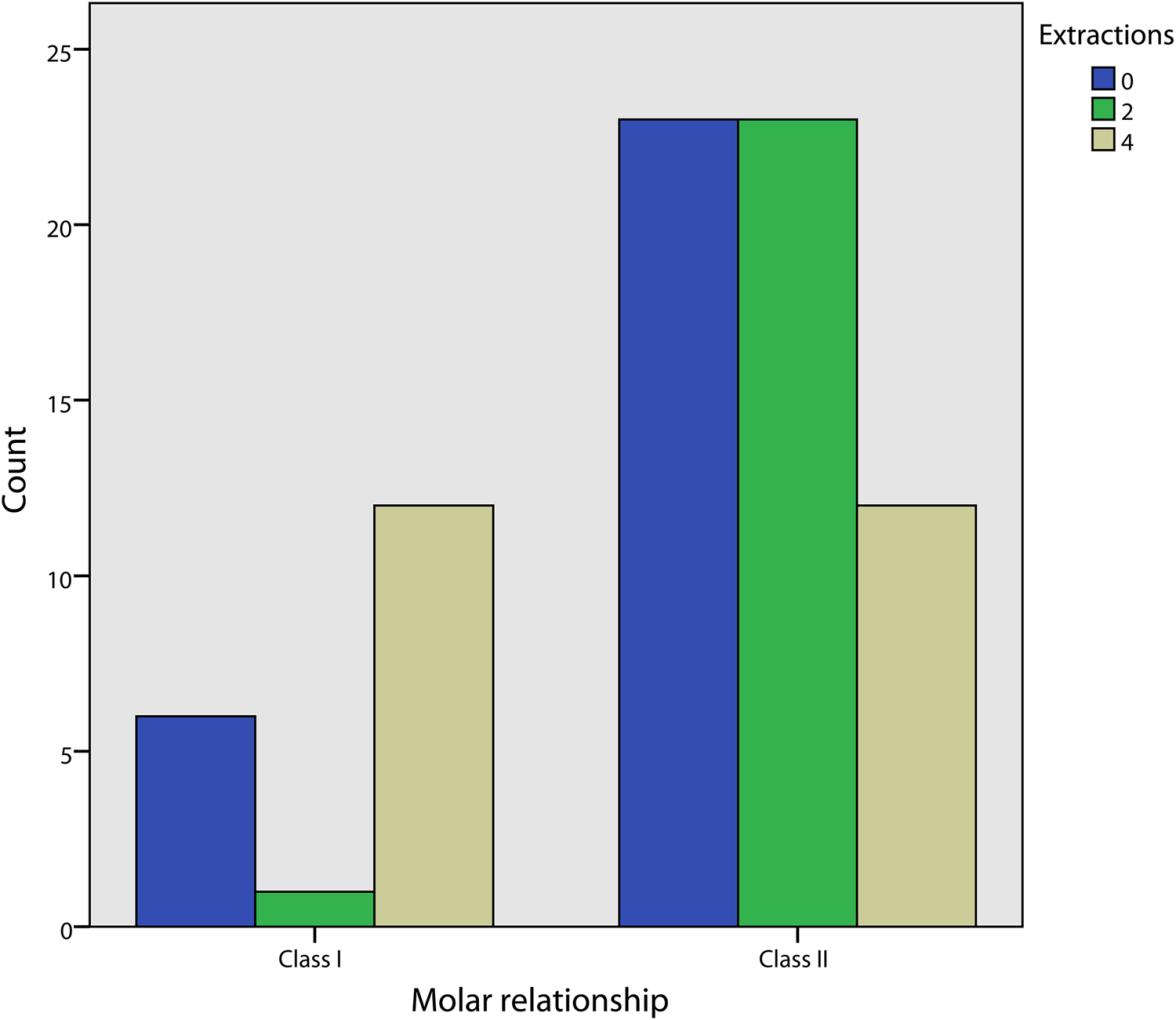
Distribution of pre treatment molar relationship

**Fig. 5:**
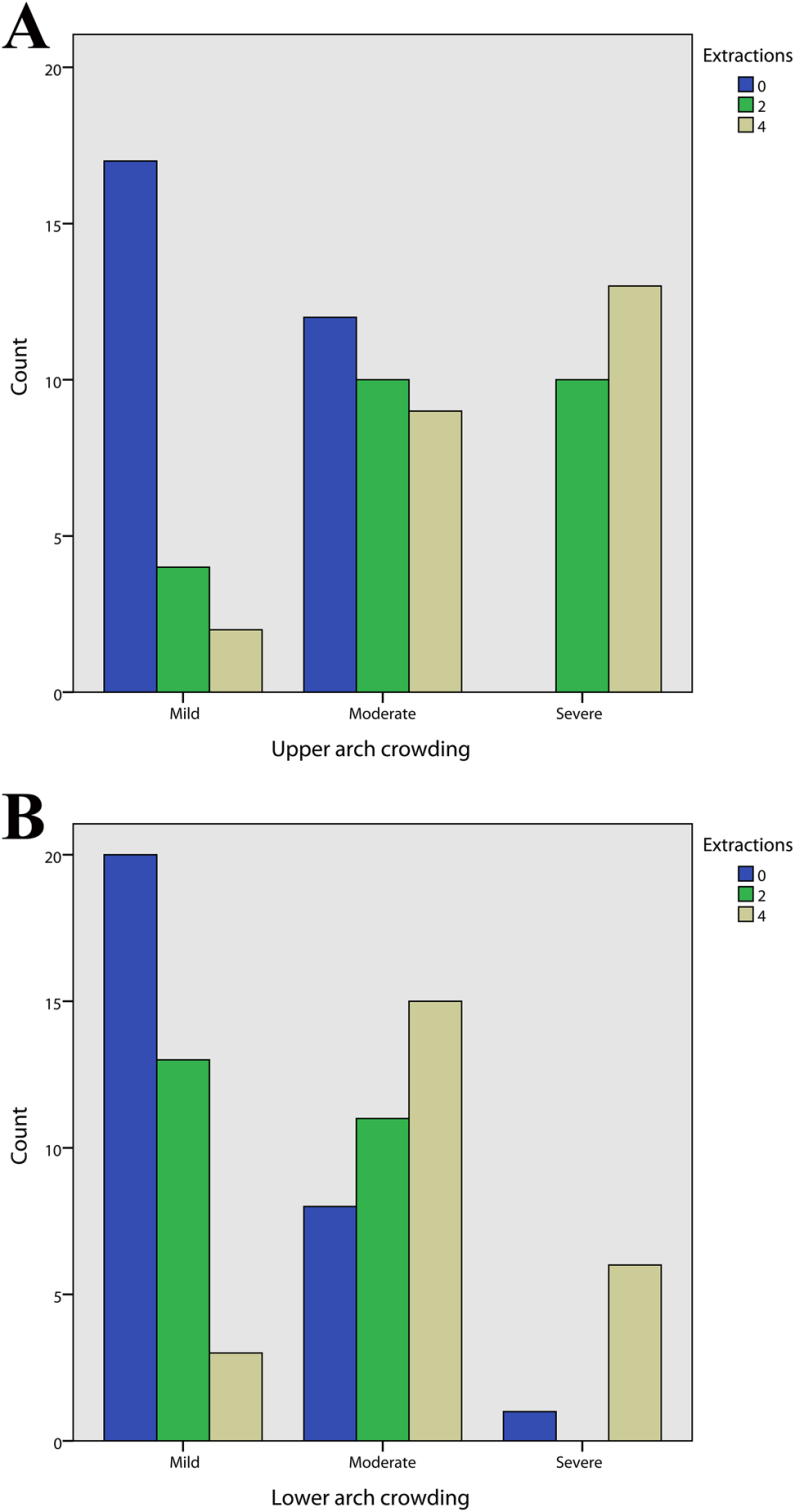
A Distribution of the amount of upper arch crowding in each group. B: Distribution of the amount of lower arch crowding in each group

**Fig. 6:**
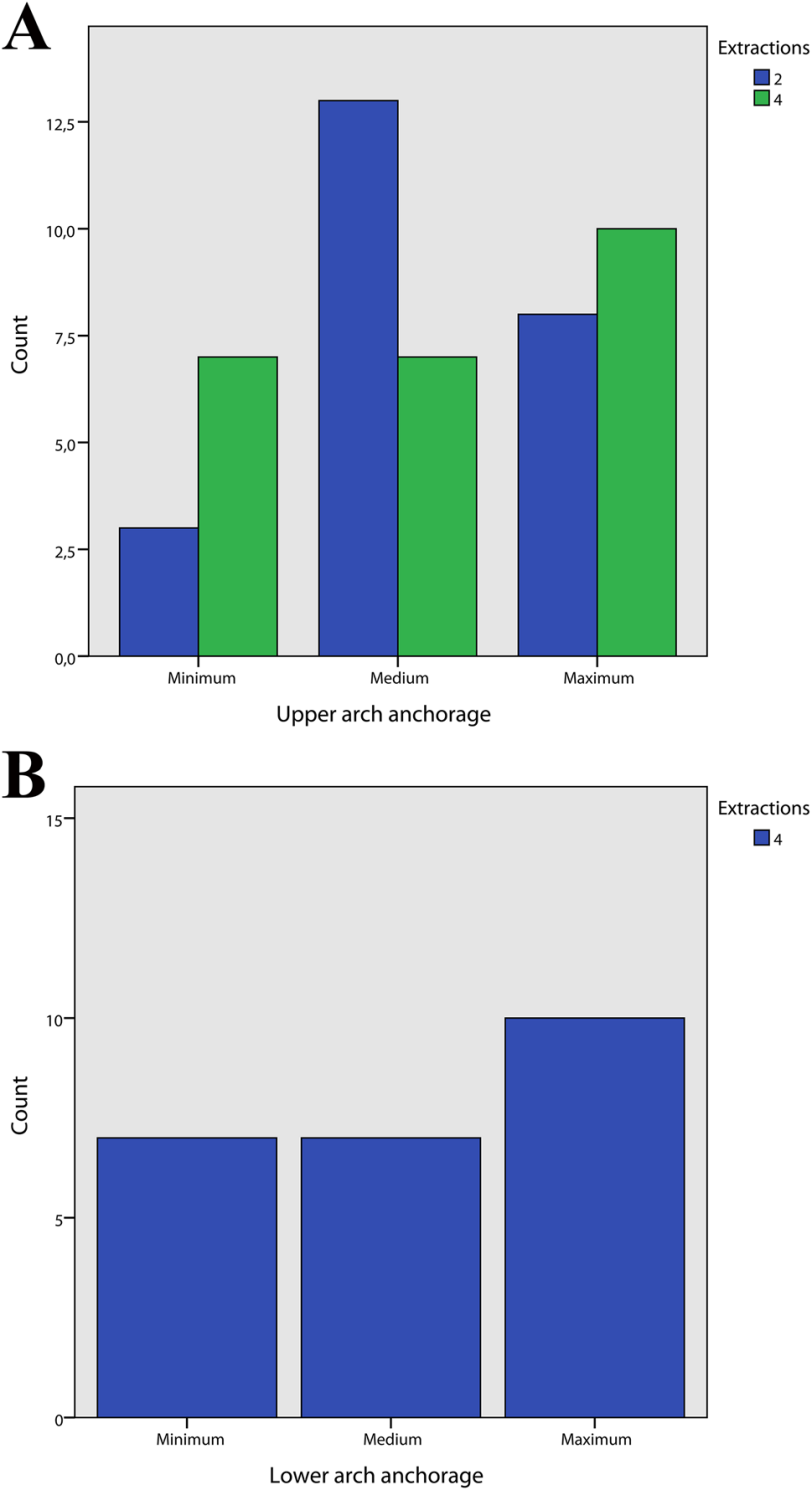
A Distribution of upper arch anchorage type in each group. B: Distribution of lower arch anchorage type in each group

The gender distribution is reported in Fig. 3, no statistically significant difference was observed between the three groups (Pearson χ^2^ = 3.64, *p* = 0.163). The distribution of mild, moderate, and severe crowding for each group and for both the upper and lower arch was calculated (Fig. 4). A significantly different distribution was observed in the control group compared to the two-extraction and the four-extraction groups for both the arches (Fisher exact for crowding in the upper arch = 31.3, *p* < 0.001; Fisher exact for crowding in the lower arch = 22.2, *p* < 0.001). Similarly, the distribution of minimum, medium, and maximum posterior anchorage for each group was determined only for the upper arch (Fig. 5) since no extractions in the lower arch were performed in the two-extractions group, and no statistically significant differences were found (Fisher exact = 3.52, *p* = 0.193) (Fig. 6).

Table 2 reports the results of the one-way ANOVA test comparing all the cephalometric variables between the three groups at T0 and T1. No differences were observed for the torque of the upper incisors at T0; however, when considering the results of the post-hoc tests (Table 3), the control group was significantly different from the other two groups regarding all other variables, but no differences were present between the two-extraction and the four-extraction groups at T0. At T1, all the cephalometric variables were different between the control group and the two-extractions group and between the control group and the four-extractions group; on the other hand, the only variables that differed at T1 between the two-extractions and the four extractions group were the Interincisive angle, the L1-GoMe angle and the Facial convexity angle (Table 3).

**Table 2:** One-way ANOVA test to compare the different variables between the three groups at T0 and at T1

*Variables*	*Levene statistic*†	*sum of sqaures between groups*	*f*	*p value*
Age T0	0.51(.601)	50.46	1.21	0.305
LU-TVL T0 (mm)	2.18(.120)	829.09	15.28**	< 0.001
LL-TVL T0 (mm)	1.08(.344)	1197.49	21.38**	< 0.001
ANB T0 (°)	0.89(.413)	94.91	7.75**	0.001
FMA T0 (°)	0.06(.941)	606.35	9.26**	< 0.001
Interincisive T0 (°)	0.43(.647)	717.63	4.42*	0.015
L1-GoMe T0 (°)	0.96(.388)	1617.32	18.96**	< 0.001
U1-ANSPNS T0 (°)	0.34(.714)	229.30	2.56	0.084
Facial convexity T0 (°)	5.56(.006)	1333.09	15.48**	< 0.001
LU-TVL T1 (mm)	0.93(.399)	826.31	18.38**	< 0.001
LL-TVL T1 (mm)	1.18(.312)	1.12	20.25**	< 0.001
ANB T1 (°)	0.26(.773)	86.71	10.43**	< 0.001
FMA T1 (°)	0.29(0.752)	301.09	3.73*	0,029
Interincisive T1	0.42(0.658)	2306.58	20.28**	< 0.001
L1-GoMe T1 (°)	2.75(.070)	3724.10	39.81**	< 0.001
U1-ANSPNS T1 (°)	2.27(.111)	520.43	7.77**	0.001
Facial convexity T1 (°)	9.20(.000)	1730.27	25.75**	< 0.001

LS-TVL: the orthogonal distance between the most protruding point of the upper lip (LU) and the true vertical line (TVL); LL-TVL: the orthogonal distance between the most protruding point of the lower lip (LL) and the TVL. The TVL was drawn tracing a perpendicular line to the Frankfurt plane passing through Na. FMA: the angle formed by the intersection of the Frankfurt plane with the mandibular plane passing through gonion (Go) and menton (Me); ANB: the angle between the A point (supra-spinal point of the maxilla). The nasion point (Na), and the B point (submental point of the jaw). Interincisal angle; the angle resulting by the intersection of the plane passing through the long axis of the upper central incisor and one passing through the long axis of the lower central incisor; U1-ANSPNS: the inclination of the upper incisor the angle between the long axis of the upper incisor and the palatal passing through ANS and PNS; L1-GoMe: the inclination of the lower incisor results of the angle between the long axis of the lower incisor and the mandibular; facial convexity: the angle obtained by connecting the glabella point (G) located on the foremost point of the forehead. Sn and pogonion cutaneous point (PgC)

^†^Levene statistic (*p* value)

^*^Statistically significant with *p* < 0.05

^**^Statistically significant with *p* < 0.01

**Table 3:** Post-hoc pairwise comparisons between all the three groups for the cephalometric variables measured at T0 and at T1

*Variables*	*Number of extractions*	*Compared group*	*Differences between means*	*SD*	*p value*	*Confidence level 95%*	
						*Lower*	*Upper*
LU-TVL T0 (mm)	0	2	6.99**	1.44	< 0.001	3.56	10.44
	0	4	6.52**	1.44	< 0.001	3.09	9.96
	2	4	− 0.47	1.50	0.947	− 4.07	3.12
LL − TVL T0 (mm)	0	2	8.86**	1.46	< 0.001	5.37	12.35
	0	4	7.18**	1.46	< 0.001	3.69	10.68
	2	4	− 1.68	1.53	0.518	− 5.33	1.98
ANB T0 (°)	0	2	− 2.65**	0.68	0.001	− 4.28	− 1.02
	0	4	− 1.60	0.68	0.055	− 3.24	0.03
	2	4	1.05	0.71	0.314	− 0.66	2.75
FMA T0 (°)	0	2	− 5.35**	0.91	0.003	− 9.12	− 1.57
	0	4	− 6.16**	0.91	0.001	− 9.93	− 2.38
	2	4	− 0.81	0.95	0.876	− 4.76	3.14
Interincisive T0 (°)	0	2	− 7.10*	2.48	0.015	− 13.04	− 1.15
	0	4	− 5.05	2.48	0.112	− 10.99	0.89
	2	4	2.05	2.60	0.712	− 4.17	8.27
U1 − ANSPNS T0 (°)	0	2	3.90	1.84	0.095	− 0.52	8.32
	0	4	0.42	1.84	0.971	− 3.99	4.84
	2	4	− 3.47	1.93	0.178	− 8.09	1.15
L1-GoMe T0 (°)	0	2	− 9.88**	1.80	< 0.001	− 14.19	− 5.57
	0	4	− 8.98**	1.80	< 0.001	− 13.44	− 4.67
	2	4	0.90	1.88	0.882	− 3.60	5.41
Facial convexity T0 (°)	0	2	− 6.57**	1.81	0.002	− 10.90	− 2.24
	0	4	− 9.79**	1.81	< 0.001	− 14.12	− 5.45
	2	4	− 3.22	1.89	0.213	-7.75	− 1.31
LU-TVL T1 (mm)	0	2	7.34**	1.30	< 0.001	4.21	10.47
	0	4	6.01**	1.30	< 0.001	2.88	9.14
	2	4	− 1.33	1.37	0.597	− 4.60	1.94
LL-TVL T1 (mm)	0	2	8.69**	1.45	< 0.001	5.22	12.16
	0	4	6.71**	1.45	< 0.001	3.24	10.18
	2	4	− 1.98	1.52	0.397	− 5.61	1.65
ANB T1 (°)	0	2	− 2.57**	0.56	< 0.001	− 3.91	− 1.22
	0	4	− 1.25	0.56	0.074	− 2.60	0.09
	2	4	1.31	0.59	0.071	− 0.09	2.72
FMA T1 (°)	0	2	− 4.57*	1.75	0.030	− 8.76	− 0.37
	0	4	− 3.35	1.75	0.143	− 7.54	0.85
	2	4	1.83	1.84	0.785	− 3.17	5.61
Interincisive T1 (°)	0	2	− 5.03*	2.08	0.047	− 10.01	− 0.06
	0	4	− 13.21**	2.08	< 0.001	− 18.19	− 8.23
	2	4	− 8.18**	2.18	0.001	− 13.39	− 2.97
U1-ANSPNS T1 (°)	0	2	5.52**	1.61	0.003	1.67	9.38
	0	4	5.30**	1.61	0.004	1.45	9.16
	2	4	− 0.22	1.69	0.991	− 4.25	3.81
L1-GoMe T1 (°)	0	2	− 16.40**	1.89	< 0.001	− 20.91	− 11.88
	0	4	− 10.85**	1.89	< 0.001	− 15.36	− 6.33
	2	4	5.55*	1.97	0.017	0.83	10.27
Facial Convexity T1 (°)	0	2	− 6.61**	1.60	< 0.001	− 10.43	− 2.78
	0	4	− 11.36**	1.60	< 0.001	− 15.19	− 7.53
	2	4	− 4.75*	1.67	0.016	− 8.76	− 0.75

^*^Statistically significant with *p* < 0.05

^**^Statistically significant with *p* < 0.01

LS-TVL: the orthogonal distance between the most protruding point of the upper lip (LU) and the true vertical line (TVL); LL-TVL: the orthogonal distance between the most protruding point of the lower lip (LL) and the TVL. The TVL was drawn tracing a perpendicular line to the Frankfurt plane passing through Na. FMA: the angle formed by the intersection of the Frankfurt plane with the mandibular plane passing through gonion (Go) and menton (Me); ANB: the angle between the A point (supra-spinal point of the maxilla), the nasion point (Na), and the B point (submental point of the jaw). Interincisal angle; the angle resulting by the intersection of the plane passing through the long axis of the upper central incisor and one passing through the long axis of the lower central incisor; U1-ANSPNS: the inclination of the upper incisor the angle between the long axis of the upper incisor and the palatal passing through ANS and PNS; L1-GoMe: the inclination of the lower incisor results of the angle between the long axis of the lower incisor and the mandibular. Facial convexity: the angle obtained by connecting the glabella point (G), located on the foremost point of the forehead, Sn and pogonion cutaneous point (PgC)

The results of the one-way ANOVA test to compare the T1-T0 variation for all the different cephalometric variables between the three groups are reported in Table 4: a statistically significant difference was observed for FMA, the Interincisive angle, the U1-ANSPNS angle, and the L1-GoMe angle. The results of the post-hoc tests for this comparison are reported in Supplementary file 1.

**Table 4:** One-way ANOVA test to compare the T1–T0 variation of all the cephalometric variables between the three groups

*Variables* *Δ (T1–T0)*	*Levene statistic*†	*Sum of sqaures between groups*	*F*	*p value*
LU-TVL (mm)	0.129(0.879)	9	0.22	0.802
LL-TVL (mm)	0.045(0.956)	2.98	0.63	0.939
ANB (°)	0.172(0.842)	1.73	0.30	0.742
FMA (°)	3,300(0.042)	107.68	4.97**	0.009
Interincisive (°)	0.732(0.485)	1422.48	7.86**	0.001
U1-ANSPNS (°)	1.102(0.277)	318.07	4.01*	0.022
L1-GoMe (°)	0.788(0.458)	577.10	7.11**	0.001
Facial convexity (°)	1.37(0.277)	40.10	0.94	0.395

^†^Levene statistic (*p* value)

^*^Statistically significant with *p* < 0.05

^**^Statistically significant with *p* < 0.01

LS-TVL: the orthogonal distance between the most protruding point of the upper lip (LU) and the true vertical line (TVL); LL-TVL: the orthogonal distance between the most protruding point of the lower lip (LL) and the TVL. The TVL was drawn tracing a perpendicular line to the Frankfurt plane passing through Na. FMA: the angle formed by the intersection of the Frankfurt plane with the mandibular plane passing through gonion (Go) and menton (Me); ANB: the angle between the A point (supra-spinal point of the maxilla), the nasion point (Na), and the B point (submental point of the jaw). Interincisal angle; the angle resulting by the intersection of the plane passing through the long axis of the upper central incisor and one passing through the long axis of the lower central incisor; U1-ANSPNS: the inclination of the upper incisor the angle between the long axis of the upper incisor and the palatal passing through ANS and PNS; L1-GoMe: the inclination of the lower incisor results of the angle between the long axis of the lower incisor and the mandibular. Facial convexity: the angle obtained by connecting the glabella point (G), located on the foremost point of the forehead,Sn and pogonion cutaneous point (PgC)

The changes of the cephalometric variables between T0 and T1 within every group were evaluated using a paired T-test or a Wilcoxon signed-rank test, whose results are reported in Table 5. A significant reduction of nearly 2° of the FMA angle was observed in the four-extraction group. The interincisive angle was significantly reduced in the two-extraction group, while it was increased in the four-extraction groups. The lower incisors were significantly tilted forward of 5° in the two-extraction group.

**Table 5:** Paired samples *T*-test and Wilcoxon signed rank test for comparing differences between T1 and T0 in all the three groups

*Variables*	*Number of extractions*	*Mean*	*SD*	*t*	*p value*	*Confidence level 95%*	
						*Lower*	*Upper*
LU-TVL (mm)	0	− 0.19	5.35	− 0.187	0.853	− 2.33	1.85
	2	− 0.53	3.85	− 0.67	0.509	− 2.15	1.10
	4	0.33	3.95	0.41	0.685	− 1.34	2.00
LL-TVL (mm)	0	0.03	5.36	0.035	0.973	− 2.00	2.07
	2	0.20	4.69	0.21	0.833	− 1.78	2.19
	4	0.51	4.40	0.57	0.577	− 1.35	2.37
ANB (°)	0	−	−	− 1.29†	0.198	−	−
	2	− 0.40	1.72	− 1.14	0.266	− 1.12	2.71
	4	−	−	− 1.84†	0.065	−	−
FMA (°)	0	0.80	3.90	1.10	0.279	− 0.68	7.70
	2	0.02	2.24	0.04	0.968	− 0.93	3.61
	4	− 2.01	3.35	− 2.94**	0.007	− 3.42	3.11
Interincisive (°)	0	− 3.21	8.98	− 1.92	0.064	− 6.63	11.20
	2	− 5.28	8.90	− 2.90**	0.008	− 9.04	10.85
	4	4.95	10.66	2.27*	0.033	0.45	22.75
U1-ANSPNS (°)	0	2.27	6.44	1.90	0.068	− 0.18	14.10
	2	0.65	5.31	0.59	0.557	− 1.59	9.07
	4	− 2.60	6.02	− 1.83	0.081	− 5.55	9.88
L1-GoMe (°)	0	− 1.74	7.08	− 1.32	0.197	− 4.43	8.30
	2	− 4.78	5.76	4.06**	< 0.001	2.34	17.0
	4	0.13	6.02	0.11	0.917	− 2.41	11.14
Facial convexity (°)	0	− 0.25	3.98	− 0.33	0.743	− 1.76	9.20
	2	− 0.21	5.78	− 0.18	0.862	− 2.65	2.71
	4	1.33	3.98	1.64	0.115	− 0.35	2.56

^†^*Z* statistic from Wilcoxon test

^*^Statistically significant with *p* < 0.05

^**^Statistically significant with *p* < 0.01

LS-TVL: the orthogonal distance between the most protruding point of the upper lip (LU) and the true vertical line (TVL); LL-TVL: the orthogonal distance between the most protruding point of the lower lip (LL) and the TVL. The TVL was drawn tracing a perpendicular line to the Frankfurt plane passing through Na. FMA: the angle formed by the intersection of the Frankfurt plane with the mandibular plane passing through gonion (Go) and menton (Me); ANB: the angle between the A point (supra-spinal point of the maxilla). The nasion point (Na), and the B point (submental point of the jaw). Interincisal angle; the angle resulting by the intersection of the plane passing through the long axis of the upper central incisor and one passing through the long axis of the lower central incisor; U1-ANSPNS: the inclination of the upper incisor the angle between the long axis of the upper incisor and the palatal passing through ANS and PNS; L1-GoMe: the inclination of the lower incisor results of the angle between the long axis of the lower incisor and the mandibular. Facial Convexity: the angle obtained by connecting the glabella point (G). located on the foremost point of the forehead.Sn and pogonion cutaneous point (PgC)

To evaluate the influence that the upper and lower incisors positions, the number of extractions, the grade of crowding, and the type of anchorage have on the profile, linear regressions were made. (Tables 6, 7, 8, and 9). These tests showed that the changes in LU-TVL and LL-TVL were mostly influenced by the type of anchorage used, in particular by the medium and minimum anchorage (Tables 6 and 7). Similarly, the facial convexity angle was mostly influenced by the type of anchorage, especially when considering the predictors of the upper arch (Table 8), even if there was a statistically significant impact of minimum and maximum anchorage in the lower arch (Table 9). On the other hand, when considering in the model for the facial convexity angle (which, of course, is relative to the entire facial profile) the predictors related to the lower arch, a significant effect of the two-extractions pattern along with the type of anchorage was observed (Table 9).

**Table 6:** Linear regressions of the effect of upper incisor torque, extraction pattern, amount of crowding, and type of anchorage (upper arch) on the position of the upper lip (LU-TVL) at T1

*Variables*	*Coefficient*	*Standard error*	*t*	*p value*
*Constant*	0.84	10.76	0.08	0.938
*U1-ANSPNS*	0	0.09	1.99	0.051
*2 Extractions*	− 1.33	1.38	− 0.98	0.336
*Moderate crowding*	− 0.55	1.37	− 0.40	0.688
*Severe crowding*	− 0.44	1.72	− 0.25	0.801
*Minimum anchorage*	− 2.42	1.38	− 1.24	0.220
*Medium anchorage*	− 5.06	1.87	− 2.71**	0.009
*Maximum anchorage*	− 5.87	1.77	− 3.31**	0.001

^*^Statistically significant with *p* < 0.05

^**^Statistically significant with *p* < 0.01. LU-TVL: the orthogonal distance between the most protruding point of the upper lip (LU) and the TVL. The TVL was drawn tracing a perpendicular line to the Frankfurt plane passing through Na; U1-ANSPNS: the inclination of the upper incisor the angle between the long axis of the upper incisor and the palatal passing through ANS and PNS

Model: *R* = 0.64, *R*^2^ = 0.42, *p* < 0.001, Durbin Watson statistics = 1.98

**Table 7:** Linear regressions of the effect of lower incisor torque, extraction pattern, amount of crowding, and type of anchorage on the position of the lower lip (LL-TVL)

*Variables*	*Coefficient*	*Standard error*	*t*	*p value*
*Constant*	7.81	10.4	0.75	0.455
*L1-GoMe*	0.12	0.09	1.31	0.195
*2 extractions*	− 1.37	1.40	− 0.98	0.332
*Moderate crowding*	0.46	1.32	0.04	0.727
*Severe crowding*	− 0.60	1.39	− 0.43	0.667
*Minimum anchorage*	− 2.19	1.92	− 1.14	0.258
*Medium anchorage*	− 4.97	1.83	− 2.71**	0.008
*Maximum anchorage*	− 5.81	1.72	− 3.38**	0.001

*R* = 0.62, *R*^2^ = 0.39,* p* < 0.001, Durbin Watson statistics = 1.70. LL-TVL: the orthogonal distance between the most protruding point of the lower lip (LU) and the TVL. The TVL was drawn tracing a perpendicular line to the Frankfurt plane passing through Na; L1-GoMe: the angle between the long axis of the lower incisor and the mandibular plane passing through the menton and gonion points

^*^Statistically significant with *p* < 0.05

^**^Statistically significant with *p* < 0.01

**Table 8:** Linear regressions of the effect of upper incisor torque, extraction pattern, amount of crowding, and type of anchorage (upper arch) on the position of the facial convexity angle (GSnPgC)

*Variables*	*Coefficient*	*Standard error*	*t*	*p value*
*Constant*	144.7	13.9	10.41**	< 0.001
*U1-ANSPNS*	0.05	0.122	0.37	0.694
*2 Extractions*	− 4.31	1.78	− 2.43*	0.018
*Moderate crowding*	2.52	1.78	1.27	0.210
*Severe crowding*	1.86	2.23	0.835	0.407
*Minimum anchorage*	9.44	2.53	3.73**	< 0.001
*Medium anchorage*	12.61	2.42	5.03**	< 0.001
*Maximum anchorage*	9.14	2.29	3.99**	< 0.001

Model: *R* = 0.65, *R*^2^ = 0.42, *p* < 0.001, Durbin Watson statistics = 1.93. Facial convexity angle: the angle obtained by connecting the glabella point (G), located on the foremost point of the forehead, subnasale (Sn) and pogonion cutaneous point (PgC); U1-ANSPNS: the inclination of the upper incisor the angle between the long axis of the upper incisor and the palatal passing through ANS and PNS

^*^Statistically significant with *p* < 0.05

^**^Statistically significant with *p* < 0.01

**Table 9:** Linear regressions of the effect of lower incisor torque, extraction pattern, amount of crowding, and type of anchorage on the position of the facial convexity angle (GSnPgC)

*Variables*	*Coefficient*	*Standard error*	*t*	*p value*
*Constant*	152.87	8.815	17.34**	< 0.001
*L1-GoMe*	− 0.02	0.107	− 0.15	0.883
*2 extractions*	7.49	2.43	3.08**	0.003
*4 extractions*	− 1.19	6.35	− 0.19	0.851
*Moderate crowding*	− 1.70	1.61	− 1.05	0.296
*Severe crowding*	− 1.37	2.88	− 0.48	0.635
*Minimum anchorage*	14.08	6.49	2.17*	0.034
*Medium anchorage*	12.73	6.53	1.95	0.055
*Maximum anchorage*	13.91	6.41	2.17*	0.033

Model: *R* = 0.66, *R*^2^ = 0.44, *p* < 0.001, Durbin Watson statistics = 1.81. Facial Convexity angle: the angle obtained by connecting the glabella point (G), located on the foremost point of the forehead, subnasale (Sn) and pogonion cutaneous point (PgC); the TVL was drawn tracing a perpendicular line to the Frankfurt plane passing through Na; U1-GoMe: the angle between the long axis of the lower incisor and the mandibular plane passing through the menton and gonion points

^*^Statistically significant with *p* < 0.05

^**^Statistically significant with *p* < 0.01

## Discussion

The results of the present research, which outlined the absence of significant aesthetic differences between the groups treated without extraction or with two or four extractions, were supported by some of the existing literature. A study that evaluated long-term changes in profiles after orthodontic extractions showed that two years after treatment, the extraction and non-extraction groups were both rated positively by observers, concluding that there were no differences between groups and that all groups were perceived more favourably when compared with the initial observation [13].

Since sagittal and vertical skeletal changes may follow extraction treatment [14], in the present study, the ANB and FMA variables were evaluated at T1 and T0 to discriminate between the soft tissue changes that could be related to such skeletal changes. According to recent studies, the influence of premolar extractions on facial profile and vertical dimension is generally overestimated and should not be the main reason for non-extraction treatment [8]. However, in the present study, FMA showed significant changes in the four-extraction group, suggesting that the vertical dimension should be considered during treatment planning. Indeed, FMA showed a T1-T0 reduction of nearly 2° (Table 5) in the four-extractions group, and this effect was statistically significant when compared to the other two groups. This result is in contrast with part of the recent literature according to which it is not necessary to do extractions to increase the anterior overbite [15]. Despite the FMA changes, there were no differences for the ANB angle between the three groups, which changed but not in a statistically significant way: meaning that extractions had no effect on the sagittal skeletal variables, as supported by different authors [16, 17].

Despite the presence of T1–T0 changes in FMA, interincisive angle and upper and lower incisor torque (Table 4), no significant differences in soft tissue aesthetics were observed between the three groups. Indeed, the mean value of incisors’ torque at T1 was in a range of 110° ± 3° for the upper arch, suggesting a good control of their position at the end of treatment. Conversely, the position of the lower incisors showed a much larger variability (Table 1) that could be attributed to the different management of crowding in the three groups. The results of this study support the hypothesis that if there is a well-controlled incisor torque, the profile does not deteriorate. In fact, it is known that bucco-lingual inclination of the maxillary incisors has a major effect on profile smile attractiveness [17]. Kusnoto et al. demonstrated the presence of a strong correlation between mandibular anterior teeth location and changes in the position of the upper and lower lips [18]. Thus, the use of a correct orthodontic mechanic, which is able to maintain a proper incisor torque, is of great importance [19].

The study results, supported by the aforementioned research, clearly showed that the soft tissue changes in the extraction and non-extraction patients were comparable at the end of treatment. Previous studies related incisor retraction to lip position, assessing that the soft tissue response was different depending on the amount of retraction of the anterior teeth [20, 21]. In extraction cases, the available extraction space was almost completely used by the retraction of the anterior segment, regardless of the number of extractions [22]. In fact, some authors analysed the amount of movement necessary for the closure of extraction spaces, concluding that the incisors underwent to a greater movement than the first molars of premolar extraction groups [23]. This significant retraction could cause the loss of incisor torque if not properly controlled, leading to a worsening of profile aesthetics.

The results of the present investigation showed that the upper incisor torque was well controlled throughout the treatment in all groups (the greatest T1-T0 change being of − 2.6° for the U1-ANSPNS angle in the four-extraction group and of 4.8° for the L1-GoMe angle in the two-extractions group), and the soft tissue changes in the three groups were comparable at the end of treatment, even if with a large standard deviation.

Although no significant differences were observed between the groups in terms of profile aesthetics, when studying the relationship between soft tissues, incisor torque, crowding and anchorage through linear regressions, it was observed that: the incisors torque did not have an impact on the soft tissue profile, while the type of anchorage showed a significant interaction, and that the two-extractions pattern had a significant interaction with the facial convexity angle.

These analyses confirmed that a well-controlled incisor torque did not modify the profile aesthetics.

Moreover, the inclusion of crowding and anchorage in multiple linear regressions were a novelty compared to previous studies. Some authors stated that the prediction of soft tissue changes could be achieved by using multivariable regression analysis [24], but they analysed only some soft tissues variables and related them to the extraction pattern without considering the anchorage and the crowding, which are two of the most important variables that could modify the treatment outcome [24, 25].

**Fig. 7:**
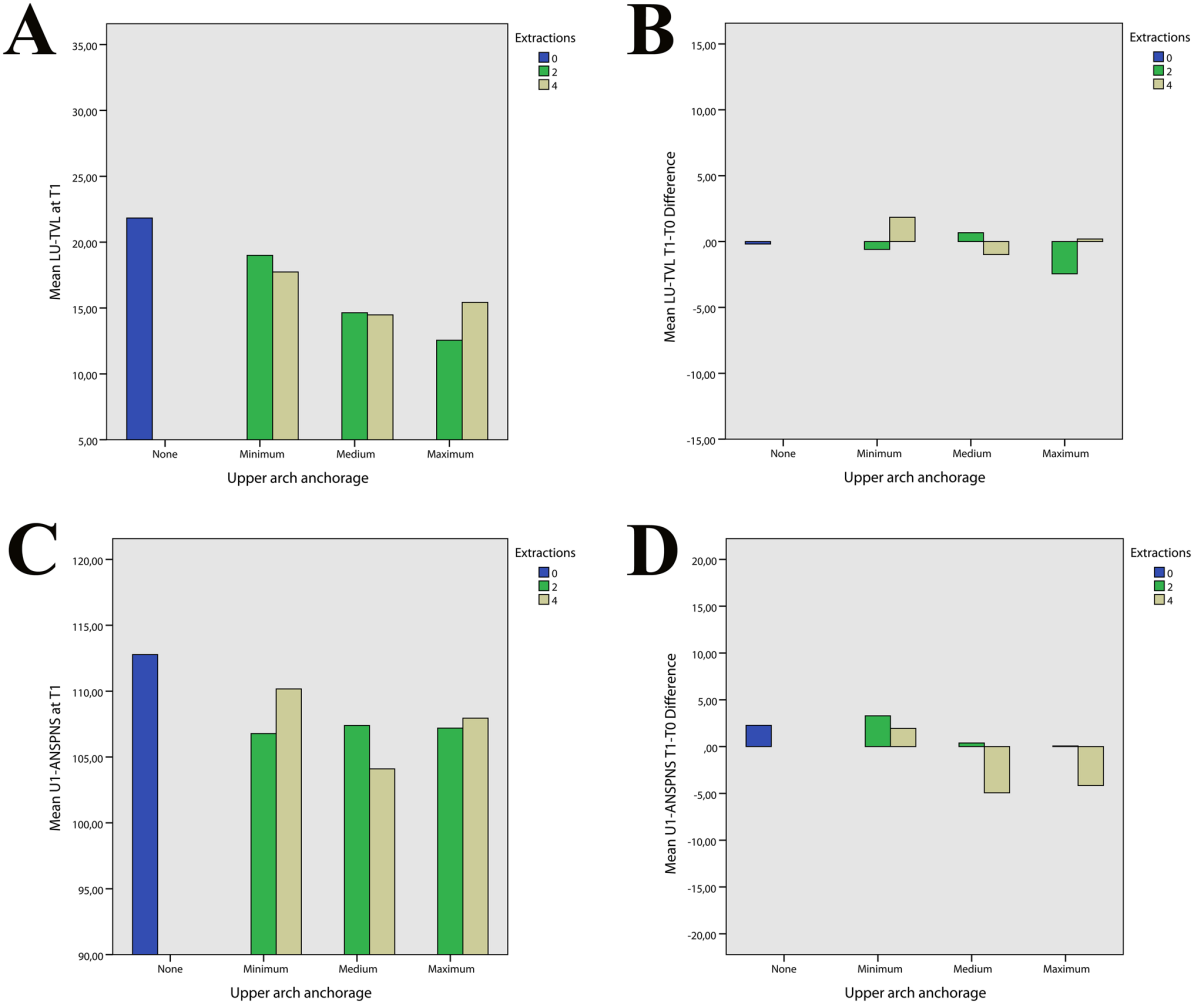
**A** Relationship between upper arch anchorage and mean of LU-TVL post treatment. **B** Relationship between upper arch anchorage and difference pre and post treatment of LU-TVL **C** Relationship between upper arch anchorage and mean of U1-ANSPNS post treatment. **D** Relationship between upper arch anchorage and difference pre and post treatment of U1-ANSPNS

**Fig. 8:**
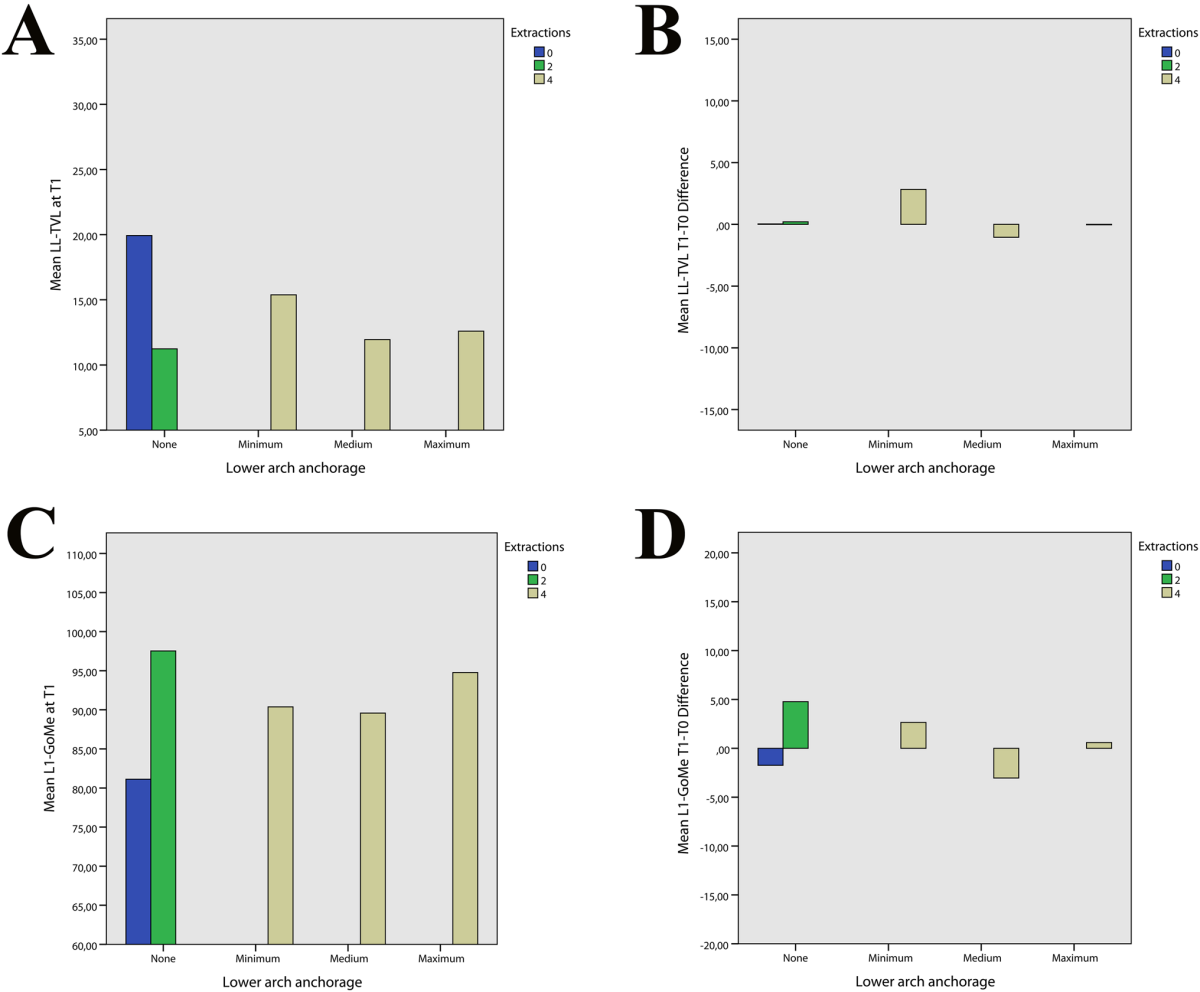
**A** Relationship between lower arch anchorage and mean of LL-TVL post treatment. **B** Relationship between lower arch anchorage and difference pre and post treatment of LL-TVL. **C** Relationship between lower arch anchorage and mean of L1-GoMe post treatment **D** Relationship between lower arch anchorage and difference pre and post treatment of L1-GoMe

Regarding the type of anchorage used, there were no differences in the upper anchorage used between the two-extraction and four-extraction groups, while the lower anchorage was classified only for four-extraction group. Looking at the LU-TVL and U1-ANSPNS values stratified by anchorage type (Fig. 7), it can be observed that the T1–T0 change of U1-ANSPNS decreased from minimum posterior anchorage to the maximum posterior anchorage in both two-extractions and four-extractions group, but that the T1–T0 change in LU-TVL was not proportional to the incisors torque. On the other hand, a more predictable behavior could be observed in the lower arch (Fig. 8). Moreover, in the present research article, the evaluation of the upper and lower lips position was assessed evaluating their distance from the TVL. Another common cephalometric method for analysing the lips position is the distance of upper and lower lips from the Ricketts’ aesthetic line. The Rickett’s aesthetic line is a cephalometric line, derived from the conjunction of the most protruding point located on the chin and the tip of nose [26]. The use of the TVL was preferred over the latter because it is well known that the human nasal cartilage changes over the years, therefore introducing a potential variability in the reproducibility of the tip of nose landmark [27, 28]. The linear regression (Tables 6, 7, 8, and 9) showed that the type of anchorage statistically influenced the facial convexity angle and the position of the upper and lower lips; in particular, while the facial convexity angle was influenced both from maximum, medium and minimum anchorage, the position of upper and lower lips was influenced only from the medium and maximum anchorage. Since in the linear regression the overall measurements of the three groups were used, the different role of the medium anchorage could be attributed to the distribution of this kind of anchorage across the three groups, and the general effect of anchorage type should be interpreted instead.

Furthermore, since extractions have always been proposed as a method of treatment for dental crowding [29], this study also evaluated the influence of the initial amount of crowding on the final aesthetic profile thanks to linear regression, observing non-significant interactions. As shown in Fig. 4, the amount of upper and lower crowding was more severe in four-extraction patients, while in two-extraction patients, there was a variable pattern. Interestingly, while the control group showed a significantly lower amount of upper and lower crowding, there were no significant differences between the four-extraction and two-extraction groups. In addition, all studied variables were comparable at T0 between the two-extraction and the four-extraction groups, suggesting that the choice of extraction pattern is a complex decision that involves many parameters, not least the clinician’s sensibility and experience.

It should be noted that all the regression models were able to explain a relatively small amount of variation, around 40%, of the dependent variables, suggesting that there are many other parameters to consider and that were not investigated in the present study, such as the phenotype of the lips and the orthodontists’ preferences. In fact, it has been observed that the correlation between the retrusion of the incisors and the flattening of the lip profile is more significant in patients with thin lips, while it is much less evident in patients with thick lips [9]. Additionally, recent research has determined that orthodontists prefer a more vestibular position of incisors rather than a lingual inclination [17], in contrast to the opinion of laypeople. Therefore, these features are surely determinants of orthodontic treatment results and should be considered for final treatment evaluation.

Finally, although care was taken to reduce the selection bias by using a rigid chronological criterion, apart from the retrospective design, the presence of subtle differences between the extraction groups and the control group limited the present investigation. However, such differences arose from the impossibility of randomising treatment choice. The need for extractions was determined by several skeletal, dental, and aesthetical considerations; therefore, it was expected that the extraction and non-extraction patients would be different from each other. It would obviously be ethically questionable to deliberately treat a patient without extraction when an extraction is required, and vice-versa. Debatable ethical issues would also arise in the case that the clinician treat a patient without an extraction who needs an extraction but refuses. These considerations were confirmed in a historical study by Odenrick et al. who compared the morphology of patients treated with and without extraction, showing differences for the dental crowding, the lengths of maxilla and mandible, and the width of teeth, which was higher in extraction patients [29].

## Conclusions

Similar soft tissue aesthetics were observed after treatment in the three groups. A statistically significant reduction in the FMA angle was observed in the four-extractions group, while a statistically significant modification of the lower incisor torque was observed in the two-extraction group. Considering the importance of soft tissue in the diagnosis of orthodontic treatment, linear multiple regression revealed that, even if there were no statistically significant differences regarding soft tissue profile, the upper and lower lip protrusion and facial convexity angle were affected by the anchorage pattern.

## Supplementary Information

Below is the link to the electronic supplementary material.

Supplementary file1 (XLSX 13 KB)

## Data Availability

The datasets generated and analyzed during the current study are available from the corresponding author on reasonable request.
